# Osteochondral Fracture of the Posterior Aspect of Lateral Femoral Condyle Presumably Caused by Tibia Pushing Up Against the Femur—A Case Report

**DOI:** 10.1155/cro/3610324

**Published:** 2025-02-07

**Authors:** Saki Inanaga, Masaki Shimizu, Shinya Yanagisawa, Keiichi Hagiwara, Masashi Kimura

**Affiliations:** ^1^Department of Orthopedic Surgery, Faculty of Medicine, Kyorin University Suginami Hospital, Tokyo, Japan; ^2^Department of Orthopedic Surgery, Asakura Sports & Rehabilitation Hospital, Gunma, Japan; ^3^Department of Orthopedic Surgery, Zenshukai Hospital, Gunma, Japan

## Abstract

A healthy 16-year-old girl presented with an osteochondral fracture of the posterior aspect of the lateral femoral condyle (LFC) following patellar dislocation. Satisfactory results were obtained with osteochondral fragment fixation and medial patellofemoral ligament (MPFL) reconstruction. To the best of our knowledge, this is only the third reported case of an osteochondral fracture of the posterior LFC. However, we believe that the mechanism of injury differed from that in the two previous cases.

## 1. Introduction

Most osteochondral fractures associated with patellar dislocation occur en bloc on the medial portion of the patella. Alternatively, the fracture of the lateral femoral condyle (LFC) is often a contusion fracture on the anterolateral portion [1]. Herein, we report a case of osteochondral fracture at the posterior LFC [2, 3]; to our knowledge, this is only the third such case reported to date. The present case differs from the two previous cases in terms of osteochondral defect morphology of the LFC and the complication of meniscal injury.

## 2. Case Presentation

A healthy 16-year-old girl (height, 151 cm; weight, 51 kg; body mass index, 22.3 kg/m^2^) presented with right knee pain and swelling. Her left leg slipped while walking, and when the patient stepped on the right leg, the knee collapsed, causing an injury. The patient had no knee dislocation or other symptoms. She had no relevant family history. Physical examination at the initial presentation demonstrated a limited range of motion (ROM) due to pain and severe knee joint swelling, and approximately 20 mL of blood was drained during knee joint aspiration.

**Figure 1:**
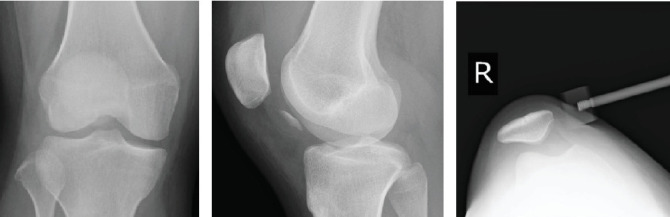
(a, b) X-ray images of a fracture fragment within the knee joint. (c) Patellar morphology is not abnormal, but the groove is slightly shallow and dislocation can be observed with external rotation stress of the knee and push to patellar.

**Figure 2:**
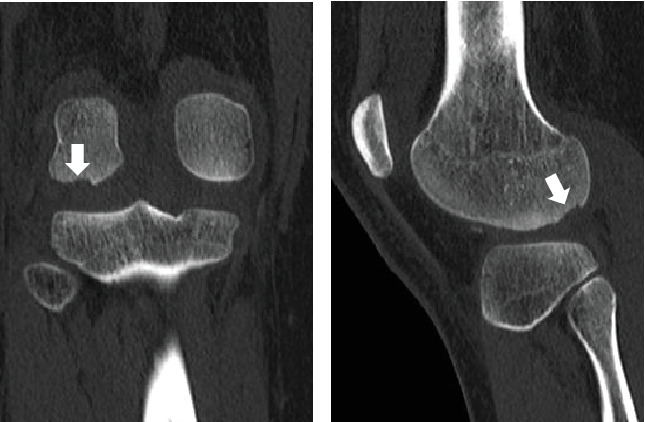
CT images of an osteochondral defect in the posterior portion of the lateral femoral condyle (LFC), as indicated with the arrow. (a) Coronal section and (b) sagittal section.

**Figure 3:**
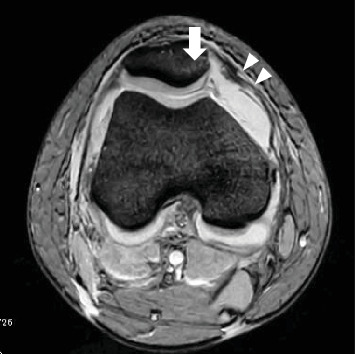
MRI images of the medial patellofemoral ligament (MPFL) injury (arrowheads) and slight bone bruises on the patella (arrow).

Radiography revealed a fractured fragment within the knee joint. The patellar morphology was not abnormal, but the groove was slightly shallow, and dislocation was observed with external rotational stress of the knee and push to the patella (Figure 1). Computed tomography (CT) revealed an osteochondral defect in the posterior portion of the LFC (Figure 2) and an avulsed bone fragment within the femoral patellar joint. No osteochondral defects were observed in the patella. No abnormalities were observed in the tibial external rotation angle (5°), and the tibial tuberosity–trochlear groove (TT–TG) distance was 7 mm. Magnetic resonance imaging (MRI) showed a medial patellofemoral ligament (MPFL) injury and slight bone bruises on the patella and LFC (Figure 3). Osteochondral fractures in the LFC were treated with reduction and fixation, and MPFL reconstruction was performed to prevent future patellar dislocation.

**Figure 4:**
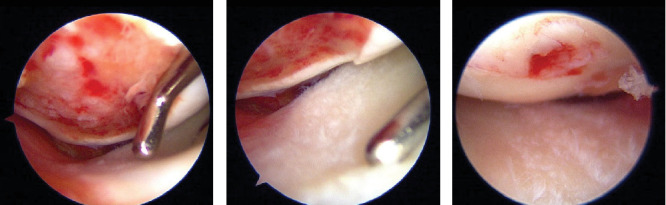
Arthroscopy images. (a) An osteochondral defect in the LFC. (b) Damage to the lateral meniscus on the femoral side. (c) Osteochondral fragment with anatomical reduction and fixing.

Arthroscopy revealed an osteochondral fragment in the femoral patellar joint and an osteochondral defect in the LFC (Figure 4(a)). The lateral meniscus was damaged on the femoral side (Figure 4(b)). The osteochondral fragment was removed from the arthroscopic portal and measured approximately 2.0 × 2.0 cm. A small lateral parapatellar incision was made, the osteochondral fragments were fixed to the defective portion of the LFC with four tibial cortical bone pegs, and additional fixation was performed with a resorbable screw (FIXSORB, Teijin, Osaka, Japan). Arthroscopy confirmed that the osteochondral fragments were anatomically reduced and fixed (Figure 4(c)). The MPFL was reconstructed using the semitendinosus muscle.

**Figure 5:**
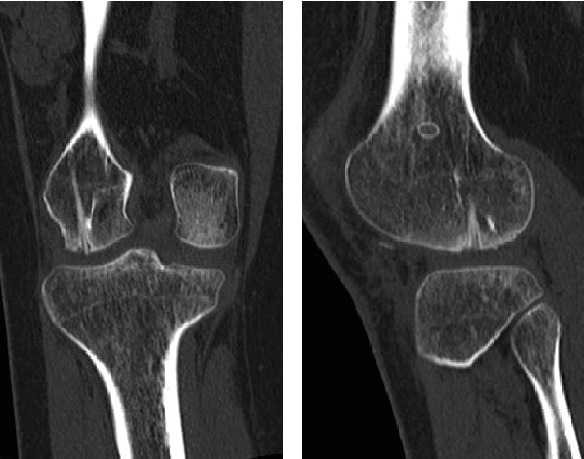
CT images showing good bony fusion and no joint step. (a) Coronal section and (b) sagittal section.

**Figure 6:**
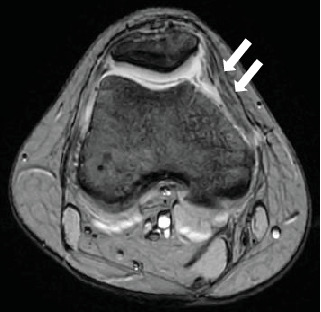
MRI image showing the reconstructed MPFL, as indicated with the arrow.

Partial weight-bearing and ROM exercises were allowed from 0° to 60° starting 1 week postoperatively, and full weight-bearing and free ROM were allowed 4 weeks postoperatively. At 1 year after surgery, the patient had no complaints of pain or discomfort in her left knee and was able to resume full activities of daily living. There was no limitation in the ROM of the knee joint or patellar instability. The mean Knee Society Score was 100. CT confirmed good bony fusion and no joint step (Figure 5). MRI provided a good visualization of the reconstructed MPFL (Figure 6).

## 3. Discussion

**Figure 7:**
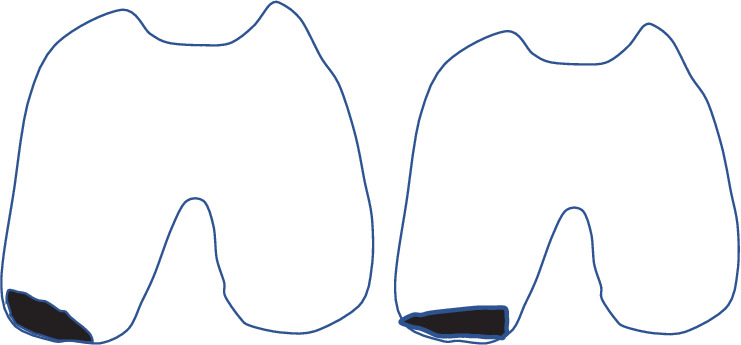
(a) In previous cases, the osteochondral defect morphology of the LFC was thickly exfoliated externally. (b) Conversely, in this case, the osteochondral defect morphology of the LFC was thickly exfoliated on the intercondylar fossa.

Traumatic patellar dislocations are more common in athletes and younger individuals [1]. Osteochondral injury of the LFC associated with patellar dislocations mostly occurs in the anterior nonweight-bearing area [4], with cartilage damage occurring in the weight-bearing area in 5%–20% of cases [1, 5]. Only two cases of osteochondral injury of the posterior portion of the LFC have been reported previously [2, 3]. Neither case had femoral torsional deformity or a history of patellar dislocation. Both cases involved female patients, in their mid-teens, with relatively minor trauma injuries sustained during dance practice or while walking. These results are similar to those observed in our case. Both previous reports also concluded that the cause of osteochondral injury was hyperflexion of the knee and collision with a dislocated patella, as the patients reported that the knee was hyperflexed at the time of injury and MRI showed no bone marrow edema of the tibial plateau. In the case reported herein, no bone edema of the tibia was observed on MRI. However, the osteochondral defect morphology of the LFC and arthroscopic findings differed from those of the two previous cases. In the previous cases, the osteochondral defect morphology of the LFC was thickly exfoliated externally, and arthroscopic findings showed no meniscal damage [2, 3]. Conversely, in this case, the osteochondral defect morphology of the LFC was thickly exfoliated on the intercondylar fossa (Figure 7), and arthroscopic findings revealed a lateral meniscus injury. The patient reported knee collapse when she stepped on the affected side when her healthy leg slipped. This led to the dislocation of the patella in the mildly flexed and externally turned position, which the patient perceived as knee collapse. The tibia was externally displaced and pushed up against the femur, causing the osteochondral defect morphology of the LFC to be thickly exfoliated on the intercondylar fossa. The lateral meniscus was considered to be trapped between the tibia and femur, resulting in injury.

However, the lack of bone edema in the tibia on MRI remains puzzling.

In this case, when dislocated, the patella may have impacted the femur, potentially causing a bone bruise, while the tibia may have pushed upward, resulting in a fracture with bone fragments. In other words, the fracture was not solely caused by the tibial thrust; therefore, the bone bruise of the tibia did not appear on MRI or CT.

When the tibia is externally displaced and thrust upward, it is normally considered that medial collateral ligament (MCL) injury may occur. However, in this case, the knee joint was flexed, resulting in minimal MCL tension. Furthermore, the patella was dislocated, which increased external instability. Thus, we concluded that MCL injury did not occur.

In this case, the treatment strategy was the fixation of the osteochondral fragment and MPFL reconstruction, considering the possibility of patellar redislocation. For fixation, four tibial cortical bone pegs were used with additional fixation using a bioabsorbable screw, as in the previous two cases.

## Data Availability

Data sharing is not applicable to this article as no datasets were generated or analysed during the current study.
